# COVID-19 and the 5G Conspiracy Theory: Social Network Analysis of Twitter Data

**DOI:** 10.2196/19458

**Published:** 2020-05-06

**Authors:** Wasim Ahmed, Josep Vidal-Alaball, Joseph Downing, Francesc López Seguí

**Affiliations:** 1 Newcastle University Newcastle upon Tyne United Kingdom; 2 Health Promotion in Rural Areas Research Group Gerència Territorial de la Catalunya Central Institut Català de la Salut Sant Fruitós de Bages Spain; 3 Unitat de Suport a la Recerca de la Catalunya Central Fundació Institut Universitari per a la Recerca a l'Atenció Primària de Salut Jordi Gol i Gurina Sant Fruitós de Bages Spain; 4 London School of Economics European Institute London United Kingdom; 5 TIC Salut Social Generalitat de Catalunya Barcelona Spain; 6 CRES & CEXS Universitat Pompeu Fabra Barcelona Spain

**Keywords:** COVID-19, coronavirus, twitter, misinformation, fake news, 5G, social network analysis, social media, public health, pandemic

## Abstract

**Background:**

Since the beginning of December 2019, the coronavirus disease (COVID-19) has spread rapidly around the world, which has led to increased discussions across online platforms. These conversations have also included various conspiracies shared by social media users. Amongst them, a popular theory has linked 5G to the spread of COVID-19, leading to misinformation and the burning of 5G towers in the United Kingdom. The understanding of the drivers of fake news and quick policies oriented to isolate and rebate misinformation are keys to combating it.

**Objective:**

The aim of this study is to develop an understanding of the drivers of the 5G COVID-19 conspiracy theory and strategies to deal with such misinformation.

**Methods:**

This paper performs a social network analysis and content analysis of Twitter data from a 7-day period (Friday, March 27, 2020, to Saturday, April 4, 2020) in which the #5GCoronavirus hashtag was trending on Twitter in the United Kingdom. Influential users were analyzed through social network graph clusters. The size of the nodes were ranked by their betweenness centrality score, and the graph’s vertices were grouped by cluster using the Clauset-Newman-Moore algorithm. The topics and web sources used were also examined.

**Results:**

Social network analysis identified that the two largest network structures consisted of an isolates group and a broadcast group. The analysis also revealed that there was a lack of an authority figure who was actively combating such misinformation. Content analysis revealed that, of 233 sample tweets, 34.8% (n=81) contained views that 5G and COVID-19 were linked, 32.2% (n=75) denounced the conspiracy theory, and 33.0% (n=77) were general tweets not expressing any personal views or opinions. Thus, 65.2% (n=152) of tweets derived from nonconspiracy theory supporters, which suggests that, although the topic attracted high volume, only a handful of users genuinely believed the conspiracy. This paper also shows that fake news websites were the most popular web source shared by users; although, YouTube videos were also shared. The study also identified an account whose sole aim was to spread the conspiracy theory on Twitter.

**Conclusions:**

The combination of quick and targeted interventions oriented to delegitimize the sources of fake information is key to reducing their impact. Those users voicing their views against the conspiracy theory, link baiting, or sharing humorous tweets inadvertently raised the profile of the topic, suggesting that policymakers should insist in the efforts of isolating opinions that are based on fake news. Many social media platforms provide users with the ability to report inappropriate content, which should be used. This study is the first to analyze the 5G conspiracy theory in the context of COVID-19 on Twitter offering practical guidance to health authorities in how, in the context of a pandemic, rumors may be combated in the future.

## Introduction

The coronavirus strains have been known since 1960 and usually cause up to 15% of common colds in humans each year, mainly in mild forms. Previously two variants of coronavirus have caused severe illnesses: severe acute respiratory syndrome (SARS) in 2002, with severe acute respiratory distress, resulting in 9.6% mortality; and Middle East respiratory syndrome in 2012, with a higher mortality rate of 34.4% [1-3]. The novel coronavirus (SARS coronavirus 2), the seventh coronavirus known to infect humans, is a positive single-stranded RNA virus that probably originated in a seafood market in Wuhan in December 2019 [4,5]. Since then, the coronavirus disease (COVID-19), named by the World Health Organization, has affected more than 2 million people worldwide, killing more than 130,000 of them [6]. The COVID-19 pandemic coincided with the launch and development of the 5G mobile network.

Compared to the current 4G networks, 5G wireless communications provide high data rates (ie, gigabytes per second), have low latency, and increase base station capacity and perceived quality of service [7]. The popularity of this technology arose because of the burst in smart electronic devices and wireless multimedia demand, which created a burden on existing networks. A key benefit of 5G is that some of the current issues with cellular networks such as poor data rates, capacity, quality of service, and latency will be solved [7]. Although there is no scientific proof, the technology is suggested to negatively affect health on certain social media channels [8].

In the first week of January, some social media users pointed to 5G as being the cause of COVID-19 or accelerating its spread. The issue became a trending topic and appeared visible to all users on Twitter within the United Kingdom. Since then, multiple videos and news articles have been shared across social media linking the two together. The conspiracy has been of such a serious nature that, in Birmingham and Merseyside, United Kingdom, 5G masts were torched over concerns associating this technology and the spread of the disease according to the British Broadcasting Corporation [9]. More concerningly, Nightingale hospital in Birmingham, United Kingdom had its phone mast set on fire [10]. This is unwelcome damage especially at a time when hospitals are required to operate with maximum efficiency.

The independent fact-checking website Full Fact noted that the conspiracy was not true and concluded that the theories given to support the 5G claims were flawed [11]. The National Health Service Director, Stephen Powis, noted in a press conference that the 5G infrastructure is vital for the wider general population who are being asked to remain at home. He noted that: “I'm absolutely outraged and disgusted that people would be taking action against the infrastructure we need to tackle this emergency” [10].

The origin of this theory demonstrates the transnational dimension to the new media landscape and the way that fake news and conspiracy theories travel. Previous research has traced the emergence of the conspiracy theory to comments made by a Belgian doctor in January 2020, linking health concerns about 5G to the emergence of the coronavirus [12]. From April 2-6, 2020, it is estimated that at least 20 mobile phone masts were vandalized in the United Kingdom alone [13]. Social media is an important information source for a subset of the population, and previous seminal research has noted the potential of Twitter for providing real time content analysis, allowing public health authorities to rapidly respond to concerns raised by the public [14]. During the unfolding COVID-19 pandemic, recent research has found that platforms such as YouTube have immense reach and can be used to educate the public [15]. Furthermore, recent research has also called for more understanding of public reactions on social media platforms related to COVID-19 [15].

The aim of this study was to analyze the 5G and COVID-19 conspiracy theory. More specifically, the research objectives were to answer the following questions: (1) who is spreading this conspiracy theory on Twitter; (2) what online sources of information are people referring to; (3) do people on Twitter really believe 5G and COVID-19 are linked; and (4) what steps and actions can public health authorities take to mitigate the spread of this conspiracy theory?

## Methods

The data set used in this article consists of 6556 Twitter users whose tweets contained the “5Gcoronavirus” keyword or the #5GCoronavirus hashtag, or were replied to or mentioned in these tweets from Friday, March 27, 2020, at 19:44 Coordinated Universal Time (UTC) to Saturday, April 4, 2020, at 10:38 UTC. Users were included in the data set if they sent a tweet during the time the data was retrieved or were mentioned or replied to in these tweets. This specific keyword and hashtag were selected, as this was the most popular and briefly became a trending topic on Twitter within the United Kingdom in early April. The network consists of a total of 10,140 tweets, which are composed of 1938 mentions, 4003 retweets, 759 mentions in retweets, 1110 replies, and 2328 individual tweets. The data was retrieved using NodeXL (Social Media Research Foundation) and the network graph was laid out using the Harel-Koren Fast Multiscale layout algorithm [16]. In interpreting the network graph, the results build upon previous seminal research, which has identified six network shapes and structures that Twitter topics tend to follow [17]. These network shapes can consist of broadcast networks, polarized crowds, brand clusters, tight crowds, community clusters, and support networks. A computer running Microsoft Windows 8 was used to retrieve data in Microsoft Excel 2010 using the professional version of NodeXL (release code: +1.0.1.428+). NodeXL uses Twitter’s search application programming interface (API). URLs were automatically expanded within NodeXL.

A number of techniques were drawn upon. First, the study used the 5Gcoronavirus keyword, which retrieved mentions of both “5Gcoronavirus” and “#5Gcoronavirus.” Second, influential users, topics, and web sources were studied, and a social network analysis of the discussion was conducted with NodeXL, a validated methodology used in previous research [18,19], which provided an understanding of the shape of the conversation. The graph’s vertices were grouped by cluster using the Clauset-Newman-Moore algorithm. Third, a manual content analysis [20] of Twitter data was conducted by removing a 10.00% sample of individual tweets (n=233/2328). Coding categories were created by exploring the data and the extracted sample was read and coded. In our content analysis, mentions were not examined because they are typically conversations between users, and retweets were excluded to avoid overpopulating the sample with similar messages. Retweets and mentions were only removed for the manual content analysis and all other analysis in the study includes them. Only English-language tweets were coded. The coding was confirmed by another author and any disagreements were discussed and resolved, which led to a 100% agreement.

Individual users have been anonymized in-line with widely cited best practices for research on Twitter [21].

## Results

### Social Network Analysis

**Figure 1:**
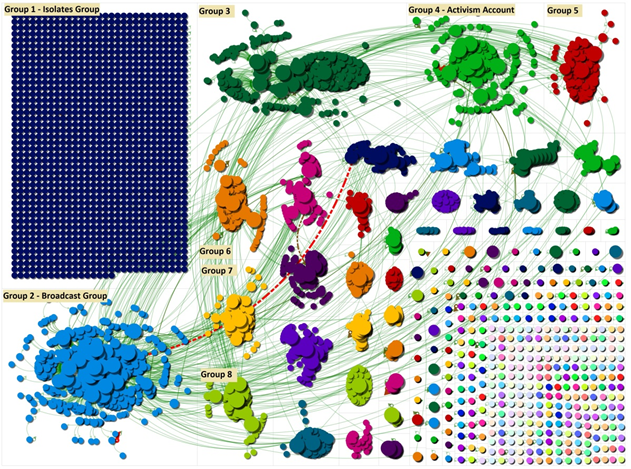
Social network graph of "5Gcoronavirus".

Figure 1 groups Twitter users in social network graph clusters. Each small color dot represents a user and a line between them represents an edge. Groups were formed around this topic based on how frequently users mentioned each other. There is an edge for each “replies-to” relationship in a tweet, an edge for each “mentions” relationship in a tweet, and a self-loop edge for each tweet that is not a “replies-to” or “mentions.” The size of the nodes has been ranked by their betweenness centrality score (BCS) [22], which measures the influence of a vertex over the flow of information between all other vertices under the assumption that information flows over the shortest paths among them.

Figure 1 highlights that a number of different groups were formed, but two large groups stand out within the cluster, which are labelled as “*Group 1 - Isolates Group*” and ““*Group 2 - Broadcast Group*.” The network shape “*Group 1 - Isolates Group*” displays users who were tweeting without mentioning one another. Isolates groups are a common network structure found in Twitter networks. Large brands, sporting events, and breaking news stories tend to have a large isolates network structure. During a sports event, for example, a large number of users may offer their view or opinion toward a team without mentioning or replying to other users forming an isolates cluster. Group 2 (labeled Group 2 - *Broadcast Group)* contains a number of Twitter accounts who would tweet that there was a link between 5G and COVID-19, which attracted retweets, giving rise to a broadcast network. Within this group, a number of influential user accounts can also be seen toward the center of the group and a circle of accounts around these. The broadcast network structure is often found in the networks for news accounts and journalists because their tweets are retweeted with high frequency. Celebrities with large followings will also tend to have a broadcast network shape. Group 4 contains the label “*activism account*” because it contained an account with the name “5gcoronavirus19,” which was set up to spread the conspiracy theory on Twitter that is further discussed in the next section.

### User Analysis

Table 1 has ranked influential users by betweenness centrality and has provided a description of the user account. The rank column orders users by their betweenness centrality score, the account description provides an outline of the type of account, the betweenness centrality column provides the raw score for each user, the follower column lists the number of followers an account had, and the NodeXL group column identifies which group Twitter users belonged to in Figure 1. The follower count is based on the amount of followers the users had during this time period.

**Table 1:** Influential users ranked by their betweenness centrality score.

Rank	Account description	Betweenness centrality score	Followers, n	Network group in Figure 1
1	Citizen	3,059,934.33	432	7
2	Citizen	3,042,916.47	12	2
3	Citizen	2,926,695.58	546	3
4	Writer	2,655,235.44	1874	2
5	5G and coronavirus dedicated activism account	2,637,433.23	383	4
6	Citizen	2,577,072.58	14	6
7	Citizen	2,354,744.84	175	2
8	Citizen	2,066,430.77	51	2
9	YouTuber	2,003,753.23	130	5
10	Donald Trump	1,380,314.74	75,916,289	4

The majority of influential users tweeting about 5G and COVID-19 consisted of members of the public sharing their views and opinions or news articles and videos supporting their cause. A key feature of the accounts was that they were actively engaged in sharing conspiracy theories; their bios included words such as “uncover” and “truth.”

User accounts ranked 1-3 appeared to be citizens who were tweeting during this time. The fourth most influential user was a writer who had over 1874 followers. Interestingly, results show that the fifth most influential account was a dedicated propaganda account (created on January 24, 2012), whose sole purpose was to raise awareness of the link between COVID-19 and 5G, and the account was named “5gcoronavirus19.” This account was in the group labeled “*activism account*” in Figure 1. The account creation date appears to be 2012, which suggests that a previously created account was converted, as Twitter allows users to change their user handle and username. The account has since been removed; however, the account bio description was “*5G causes our immune system to lower and we become more susceptible to viruses. Wuhan was the FIRST FULL 5G city! #Coronavirus caused by 5G*.” This user was in group 4 in the network graph outlined in Figure 1 and had sent a total of 303 tweets in the 7-day time period studied in this paper. Group 4 contained a total of 408 Twitter accounts. At tenth place, the president of the United States, Donald Trump, appears as an influential user; however, unlike other users in the network, Trump did not directly tweet about the link between COVID-19 and 5G. Trump appears because he is mentioned by other Twitter users related to general policy and discussion surrounding 5G. All other users within the network were actively tweeting during this time period.

The analysis reveals that there was a lack of an authority figure who was actively combating such misinformation. Twitter users that have the highest number of mentions during this time period are shown in Multimedia Appendix 1. The two most mentioned users were members of the public, and the third most mentioned user was the dedicated COVID-19 activism account mentioned previously and ranked the fifth most influential user in Table 1. Multimedia Appendix 2 lists the users that were replied to the most during this time period. The second most mentioned user was again the dedicated coronavirus activism account. This shows how the account linking 5G to COVID-19 also stimulated debate on Twitter and held power over the network because the account was both highly influential and mentioned.

### Influential English-Language Websites

In order for the conspiracy theory to spread the public needs information and a reference source. This study has identified which sources were influential during this time. Table 2 highlights the most influential websites relating to this topic during this time period. The most popular web source shared on Twitter during this time was the website known as *InfoWars*, which is a popular conspiracy theory website based in the United States. The article itself linked to several videos in which “top scientists” revealed how 5G could weaken a population’s immune system. The rank column refers to the ranking of each website based on the count column.

It can be seen that the majority of the websites can be argued as being “fake” or “alternative” news websites. The websites and information shared on Twitter can also shed light on the types of sources social media users were drawn toward. Multimedia Appendix 3 shows the most frequently occurring domains within the network. This analysis is different to that conducted in Table 2, as it identifies overall web domains that were most used in tweets rather than specific websites, showing, for instance, that YouTube appears ranked as the second most popular domain.

**Table 2:** Influential web sources.

Rank	Website title	Source	Tweets, n
1	“WATCH LIVE: CORONAVIRUS HOME SCHOOL SPECIAL & ASK THE EXPERTS! Prestigious doctors & scientists confirm 5G weakens the immune system to all viruses including Covid-19”	InfoWars [23]	38
2	“VIDEO: Former President Of Microsoft Canada, Frank Clegg: 5G Wireless IS NOT SAFE”	RayGuardNJ Electrosmog Protection [24]	31
3	“There’s A Connection Between Coronavirus And 5G”	Stillnessinthestorm [25]	20
4	“BREAKING NEWS: Slovenia Stops 5G Due to Health Risks”	5gcrisis website [26]	18
5	“CAN YOU BELIEVE THIS??”	Jeff Censored! YouTube channel. [27]	18

### Content Analysis

From the overall data set, a 10.00% sample of tweets (n=233/2328) that did not mention or reply to another user were extracted. Content analysis revealed that, of the 233 sample tweets, 34.8% (n=81) of individual tweets contained views that 5G and COVID-19 were linked, 32.2% (n=75) denounced the conspiracy theory, and 33.0% (n=77) were general tweets not expressing any personal views or opinions. Table 3 below displays the results of this coding alongside examples of tweets.

The focus was to identify the percent of pro- and anticonspiracy themes. Any other tweets would be classified as “general tweets.” It was found that 32.2% (n=75/233) of tweets were views against the conspiracy theories that were being shared. They either attacked or ridiculed those sharing such views with humor.

The second category contained tweets that were general in nature and used the “5G” keyword or hashtag in their tweets as highlighted in Table 3. This occurred in 33.0% (n=77/233) of tweets. Users may have used the keywords and hashtags for additional exposure. This theme also contained general news articles related to 5G and COVID-19. This is not surprising, as other Twitter users attempt to “link bait” on Twitter by flooding popular topics with content to obtain more viewers for their own tweets or web links.

The next category consisted of tweets that were clearly expressing views against the conspiracy or were intending to be humorous toward those linking 5G and COVID-19.

The largest category of users, with 34.8% (n=81/233) of the tweets, were engaging with and spreading information that linked COVID-19 and 5G. Anonymized tweet extracts for this theme are provided in Textbox 1.

Thus, 65.2% (n=152/233) of tweets derived from nonconspiracy theory supporters, which suggests that, although the topic attracted high volume, only a handful of users genuinely believed the conspiracy. It is also worth noting that on April 4, 2020, the media began to report that a number of 5G masts had been set on fire [8]. This coincides with the final day that we collected data, and we observed users actively encouraging other users to destroy 5G towers, as highlighted by the final three anonymized tweet extracts in Textbox 1.

**Table 3:** Content analysis of individual tweets (n=233).

Category	Theme	Example	Tweets, n (%)
1	5G and the coronavirus disease are linked	“*5G Kills! #5Gcoronavirus - they are linked! People don’t be blind to the truth!*”	81 (34.8)
2	General tweets not expressing a view or opinion	“*I have a 10AM Skype Chat on Monday, COVID-19 #5Gcoronavirus*”	77 (33.0)
3	Anticonspiracy theories or humor	“*5G is not harming or killing a single person! COVID-19 #5Gcoronavirus*”	75 (32.2)

**Anonymized tweet extracts from category 1.**

**Tweets**

“5G is the one and only Coronavirus! Radiation from it will easily wipe out the world population. Think! Why did China get rid of their 5G towers? This is why they are now free from the Corona.”

“5G volumes peaked and infected COVID-19 cases in Italy also peaked, no coincidence!”

“People must open their minds and see the truth that 5G kills!”

“I didn’t believe in all of this stuff until I read this article! [URL] Folks, please educate yourselves!”

“Make sure to SMASH THOSE 5G masts up!! #5Gcoronavirus”

“5G Towers are burning [link to video] - now what should we do with the others?”

“Hope we can see some more go down”

## Discussion

Academics have been alarmed at the rate of fake news and misinformation across social media [28-32]. Initially, social media platforms had been praised for their ability to spread liberal messages during events such as the Arab Spring [22] and during the initial launch of WikiLeaks [33]. False information has been a genuine concern among social media platforms during COVID-19, and Facebook has implemented a new feature that will inform users if they have engaged with false information [34].

One method of counteracting fake news is to be able to detect it rapidly and address it head-on at the time that it occurs. In the specific influencer analysis (in the User Analysis section), there was a lack of an authority figure who was actively combating such misinformation. This study found that a dedicated individual Twitter account set up to spread the conspiracy theory formed a cluster in the network with 408 other Twitter users. This account, at the time of analysis, had managed to send a total of 303 tweets during this specific time period before it was closed down by Twitter. In hindsight, if this account would have been closed down much sooner, this would have halted the spread of this specific conspiracy theory. Moreover, if other users who were sharing humorous content and link baiting the hashtag refrained from tweeting about the topic and instead reported conspiracy-related tweets to Twitter, the hashtag would not have reached trending status on Twitter. As more users began to tweet using the hashtag, the overall visibility increased. Public health authorities may wish to advise citizens against resharing or engaging with misinformation on social media and encourage users to flag them as inappropriate to the social media companies. Many social media platforms provide users with the ability to report inappropriate content.

A further method of counteracting misinformation is to seek the assistance of influential public authorities and bodies such as public figures, government accounts, relevant scientific experts, doctors, or journalists. A further key point to make is that the fight against misinformation should take place on the platform where it arises. This is because people will not go to a website to read the counteracting report, but they will watch a video or a memo voice sent via WhatsApp or posted on a social media platform. Public TV, newspapers, and radio stations could also seek to devote regular programs to counteract fake news by discussing conspiracy theories that were spreading at the time. It could also be argued that it is important to analyze the context of the fake news and why it is spreading. Are people afraid? Does the theory propose a risk? Any content that aims to correct misinformation should aim to dispel people’s fears.

This research set out to address four research questions that are now discussed. In regard to identifying how the conspiracy was spreading on Twitter, this article shows that a number of citizens who believed the conspiracy theory were actively tweeting and spreading it (as highlighted in Table 1). A dedicated account that was set up for the sole purpose of spreading the conspiracy theory was identified. We also identified the “humor effect” in the sense that even those users who joined the discussion to mock the conspiracy theory inadvertently drew more attention to it.

In addressing the second research objective, this paper identifies a number of influential online sources that created content aiming to show a link between COVID-19 and 5G (as highlighted in Tables 2 and 3). These consisted of the website *InfoWars,* a commercial organization selling products that protect against electromagnetic fields. A website dedicated to linking 5G to COVID-19 was also identified. Specific YouTube videos and the YouTube domain itself were also found to be influential.

The third research objective was to identify whether people really believed 5G and COVID-19 were linked, as Twitter is known to contain humorous content [16]. It was found that 34.8% (n=81/233) of individual tweets contained views that 5G and COVID-19 were linked. Although it is a low percentage, there are indeed users who genuinely believe COVID-19 and 5G are linked.

In regard to the fourth research objective, this article sought to identify and discuss potential actions public health authorities could take to mitigate the spread of the conspiracy theory. Specifically, this study found that an individual account had been set up to spread the conspiracy theory and was able to attract a following and send out many tweets. Based on our analysis of this conspiracy theory on Twitter, its spread could have been halted if the accounts set up to spread misinformation were taken down faster than they were. Public health authorities should also aim to focus on these types of accounts in combating misinformation during the current COVID-19 pandemic. In addition, an authority figure with a sizeable following could have tweeted messages against the conspiracy theory and urged other users that the best way to deal with it is to not comment on, retweet, or link bait using the hashtag. This is because when users joined the discussion to dispel, ridicule, or piggyback on the hashtag, the topic was raised to new heights and had increased visibility.

A strength of this study is that it has identified the drivers of the conspiracy theory, the content shared, and the strategies to mitigate the spread of it. Our results are likely to be of international interest during the unfolding COVID-19 pandemic. A further strength of our study is that our methodology can be applied to other conspiracy topics. A limitation of our study is that the Search API can only retrieve data from public facing Twitter accounts. Previous research has noted that certain Twitter topics are likely to contain automated accounts known as “bots” [35]; for instance, in the case of electronic cigarette (e-cigarette) tweets, research has found that social bots could be used to promote new e-cigarette products and spread the idea that they are helpful for smoking cessation [35]. A limitation of our study is that we did not identify social bot accounts; however, influential accounts in our study did not appear to display bot behavior (eg, high number of tweets posted) and appeared to display characteristics of genuine accounts. This could be inferred because certain accounts linked to their profile on other platforms such as YouTube. However, future research could seek to identify the ratio of bots to individual accounts related to conspiracy theories. A further limitation is that our content analysis was conducted on English-language tweets, and further research could seek to examine tweets in other languages. Furthermore, a limitation to our study is that, as we retrieved data using a specific keyword, our data may have excluded tweets from users who tweeted about the conspiracy during this time without using our target keyword or hashtag.

The COVID-19 pandemic has been a serious public health challenge for nations around the world. This study conducted an analysis of a conspiracy theory that threatened to potentially undermine public health efforts. We discussed key users and influential web sources during this time, and discussed potential strategies for combating such dangerous misinformation. The analysis reveals that there was a lack of an authority figure who was actively combating such misinformation, and policymakers should insist in efforts to isolate opinions that are based on fake news if they want to avoid public health damage. Future research could seek to conduct a follow-up analysis of Twitter data as the COVID-19 pandemic evolves.

## Appendix

Multimedia Appendix 1 Top mentioned users.

Multimedia Appendix 2 Top replied-to users.

Multimedia Appendix 3 Top domains.

## Abbreviations

BCS: betweenness centrality score
COVID-19: coronavirus disease
e-cigarette: electronic cigarette
SARS: severe acute respiratory syndrome
UTC: Coordinated Universal Time
