# Supplementary material for: COVID-19 and the 5G Conspiracy Theory: Social Network Analysis of Twitter Data
Source: J Med Internet Res. 2020 May 6;22(5):e19458. doi: 10.2196/19458 (PMC7205032; doi:10.2196/19458)
Supplement: Multimedia Appendix 1 [file jmir_v22i5e19458_app1.docx]

| **Rank** | **Top Mentioned in Entire Graph** | **No. of Mentions** |
| --- | --- | --- |
| 1 | Member of the Public | 155 |
| 2 | Member of the Public | 86 |
| 3 | 5G and Coronavirus Dedicated Activism Account | 85 |
| 4 | Donald Trump | 83 |
| 5 | YouTube Twitter account | 50 |
| 6 | Member of the Public | 46 |
| 7 | Author | 45 |
| 8 | Member of the Public | 45 |
| 9 | Member of the Public | 40 |
| 10 | Member of the Public | 38 |
