# Supplementary material for: COVID-19 and the 5G Conspiracy Theory: Social Network Analysis of Twitter Data
Source: J Med Internet Res. 2020 May 6;22(5):e19458. doi: 10.2196/19458 (PMC7205032; doi:10.2196/19458)
Supplement: Multimedia Appendix 2 [file jmir_v22i5e19458_app2.docx]

| **Rank** | **Top Replied-To in Entire Graph** | **Count** |
| --- | --- | --- |
| 1 | Member of the Public | 154 |
| 2 | 5G and Coronavirus Dedicated Activism Account | 95 |
| 3 | Member of the Public | 24 |
| 4 | Account Deleted was titled *#CoronaMediaHoax* | 18 |
| 5 | Author | 18 |
| 6 | Member of the Public | 17 |
| 7 | BBC News Reporter | 15 |
| 8 | Member of the Public | 14 |
| 9 | Member of the Public | 14 |
| 10 | Donald Trump | 13 |
