# Supplementary material for: COVID-19 and the 5G Conspiracy Theory: Social Network Analysis of Twitter Data
Source: J Med Internet Res. 2020 May 6;22(5):e19458. doi: 10.2196/19458 (PMC7205032; doi:10.2196/19458)
Supplement: Multimedia Appendix 3 [file jmir_v22i5e19458_app3.docx]

| **Rank** | **Top Domains in Tweet in Entire Graph** | **Count** |
| --- | --- | --- |
| 1 | twitter.com | 465 |
| 2 | youtube.com | 336 |
| 3 | transitieweb.nl | 39 |
| 4 | infowars.com | 38 |
| 5 | worldunity.me | 38 |
| 6 | rayguardnj.com | 31 |
| 7 | jdreport.com | 21 |
| 8 | stillnessinthestorm.com | 20 |
| 9 | 5gcrisis.com | 19 |
| 10 | Dailymail.co.uk | 10 |
